# Diseases of the Maxillary Bones and Their Periosteum

**Published:** 1894-05

**Authors:** Vida A. Latham

**Affiliations:** F.R.M.S.; Chicago, Ill.


### Diseases of the Maxillary Bones and their Periosteum.

Read in the Section on Dental and Oral Surgery, at the Forty-fourth Annual Meeting of the American Medical Association.

BY VIDA A. LATHAM, F.R.M.S., D.D.S , CHICAGO, ILL.

[*Continued from April Number, page* 163.]

*Diseases of the Alveolar Dental Periosteum.*—The principal one is inflammation (periodontitis), and its causes number at least seventeen, viz.: want of occlusion; malocclusion; tartar; looseness of teeth; induration of tooth-tissue; cavities of decay impinging on cementum; excess of filling material; pulpitis; extraction of pulp without hemorrhage; external irritation by forcible removal of pulp; enlarged foramen; putrescent pulp; previous periodontitis; action of medicines locally; contact with arsenic; morphin; action of medicines systemically; action of syphilis, fever, diphtheria, gout, rheumatism. Inflammation here is not different, pathologically, from any other, but it has special symptoms because of its position. Rheumatic periostitis is acutely painful; does not lead to suppuration, and varies with the weather. In mercurial periodontitis the breath is very foul, and if mercury is continued the whole surface sloughs away, together with much of the alveoli. Strumous periodontitis tends to rapid suppuration, swelling and abscess, and is less painful. Periodontitis due to phosphorus fumes is apt to end in necrosis of the entire jaw-bone.

The varieties of periodontitis are:

1. Acute, sub-acute and chronic.

2. Traumatic.

3. Absorption of roots in diseased condition of the membrane and after injuries, transplantations, replantations, etc. When the membrane is healthy, no absorption of roots of permanent teeth takes place.

4. Apical. Confined to the end of the root, following death of pulp.

5. Gingivitis. Inflammation of peridental membrane at border from constitutional causes, as ptyalism.

6. Calcic inflammation.

7. Phagedenic. A specific infectious inflammation having its beginning in the gingival border, and accompanied by destruction of the periodontium and alveolar walls. Riggs' disease. Periodontitis may be local or general, the majority of cases being the latter.

Repair of the peridental membrane is said never to take place by first intention, but always by granulation, which may begin in the tissue overlying the parts of the root, but the re-attachment creeps in from the injury where the peridental membrane is intact or from the extremity of the pocket above, and slowly covers over the denuded portions of a tooth. Sponge-grafting has been suggested to renew the gingival and lower border of the peridental membrane when lost from phagedenic periodontitis.

Periostitis, both acute and chronic, affects the jaws. The acute form is a dangerous one, as it so often runs into suppuration with consequent necrosis. It is only in the early stage that it can be recognized. The more chronic form is commonly connected with syphilis, and leads to the formation of nodes about the palate and enlargement of portions of the inferior maxilla, and it may be well to mention that many cases of persistent facial neuralgia, which are unrelieved by quinin, etc., yield to potassium iodid, and may be said to depend upon chronic periostitis or osteitis with, probably, pressure on the dental nerves.

Necrosis. This disease affects the inferior much more commonly than the superior maxilla, probably in consequence of its being less vascular. (Heath.) Beginning as periostitis from dental irritation, injury, action of specific poisons, the symptoms generally are, pain with pyrexia; part swollen, ingested; teeth raised and unable to bear any pressure. If immediately relieved by leeches, free incision, hot poultices, etc., the symptoms may subside and no further injury result; but usually pus will be found beneath the periosteum before the patient comes to you, and even then, though evacuated instantly, necrosis is usually the result. Luckily, necrosis sometimes only affects the external plate of the alveolus, so you get the teeth supported by the internal plate and you can keep them in situ; but when the entire alveolus is involved, the teeth loosened and possibly useless, they had better be removed, as they only stop up the discharge. Do not attempt to remove sequestra unless you are sure they are completely loosened, or you may inflict injury on the surrounding parts and interfere with the process of repair, and this is especially the case with children in whom the second teeth are still undeveloped. The time of separation is difficult to decide on, for we must depend on the strength of the patient, and the extent and position of sequestrum. It takes usually about six weeks to three months before large sequestra can be safely removed.

Another form of necrosis is the exanthematous, so named by Salter. This name is applied to the diseases found in young children, usually after attacks of specific fevers, especially scarlet fever and small-pox. Necrosis of portions of the alveolus of either maxilla which usually occurs on both sides alike, or even when it involves the whole thickness of the lower jaw, is now taken as one of the sequelæ of these diseases; and possibly many cases in years gone by, thought to be due to calomel, were solely due to the action of the specific poison. The repair of bone in necrosis of the upper jaw is a little different from that of the lower. No reproduction of bone takes place, the gap left in adults being permanent, though in children, the subjects of exanthematous necrosis, the granulation tissue is slowly converted into fibrous tissue, which does not as a rule ossify. In the inferior maxilla, abundant new bone is formed by the periosteum, and for a time, at least, most extensive losses are repaired. It is certain, however, that in the course of years, a great, if not a complete re-absorption of new bone thus formed takes place, the patient being left finally with very little if any support for artificial teeth. Prof. Salter has suggested that the early application of artificial teeth would tend, by use, to strengthen and maintain the permanence of the new bone; but I believe there are no facts to support this view.

The action of the fumes of phosphorus is well known to cause necrosis, but is less common now than formerly, on account of the substitution of red amorphous phosphorus for the yellow variety. It only occurs when the teeth are carious and the jaws in an unhealthy state. Those with carious teeth who work with phosphorus are always affected, and the necrosis is violent and rapid. The disease may begin as osteo-periostitis which rapidly ends in necrosis, or it may begin by osteo-plastic inflammation, swelling, profuse and offensive suppuration of the soft parts, attended with pyrexia. These symptoms are sometimes followed by gangrene, erysipelas and death. Sometimes the removal of the sequestrum is followed by recovery. The whole jaw is usually involved, the callus lying between the sequestra. The notable point to be observed in phosphorus necrosis is the peculiar deposit of pumice-like bone which takes place upon the sequestra. This no doubt comes from the periosteum, although so closely adjoining the sequestrum as to be almost always brought away with it; and though resembling true bone in some particulars it is a little lower developed form of bone. A deposit closely allied to this, however, has been noticed in cases in which there was no phosphorus involved, and it would appear that, in some instances, possibly of rheumatic origin, the deposit of new bone partakes of this character.

The action of mercury can be briefly mentioned here: it injures the teeth in two ways; by salivation and by the graver injury, the production of honeycombed teeth, which is often confused with the effects of inherited syphilis. Honeycombed teeth are the result of mercurial treatment during the period when the enamel is being calcified (J. Hutchinson, *Path. Soc. Trans.*, Vol. XXVI, 1875, pp. 235 et seq.) The two affections have been discussed by Mr. Hutchinson together, and because they are coincident with ophthalmic changes and the question of mercury occurs in both, much confusion has arisen. Below is briefly contrasted and tabulated these conditions:

| HONEYCOMBED. | SYPHILITIC. |
|---|---|
| 1. Are peculiar only in the latest formed tissue, the last layer of enamel. | Are most peculiar in the earliest formed tissue, the last formed enamel being usually perfect. |
| 2. Perfect at birth, become affected after a year or so. | Are imperfect from earliest germ stage. |
| 3. Are coincident with lamellar cataract, infantile fits, and mercurial treatment. | Are coincident with interstitial keratitis, inherited syphilis, and have nothing to do with mercurial treatment. |
| 4. The teeth that suffer most are the first permanent molars. | The teeth that suffer most are upper incisors. |
| 5. The general form of the teeth is unaltered. | The form of the teeth is profoundly modified. |

Mercury, as a rule, on the deciduous teeth is a lasting injury, but on the periosteum in adult life it is transient unless long continued. In mild cases, in addition to periostitis, there is a profuse flow of saliva, a mercurial fœtor of the breath, with later on swelling and sloughing of the alveolus and gums, upon which in severe cases a whitish membrane appears; this is at first lightly attached, but later is adherent, and it is *underneath this* that ulceration of the gums takes place. The teeth soon loosen and are lost, and in very bad and neglected cases the whole surface sloughs away, together with much of the alveoli, and necrosis of the whole jaw may result, and we have mercurial stomatitis.

Periostitis due to rheumatism will very likely pass away, especially if much affected by the weather, but it is often difficult to persuade a patient to retain a dead tooth which keeps up this periodical irritation. For these cases, what treatment can be given other than systemic remedies, counter-irritation, warmth and patience?

Periostitis due to syphilis or struma is not so easily disposed of, for it usually progresses to the worst degree. Salivary calculus, if removed in time, will scarcely ever cause dangerous periostitis, but the serumal is a great deal more likely to set up and prolong irritation to such a degree as to cause exostosis and its concomitants; and as we can seldom remove it unless by extracting the tooth and scraping the deposit from the ends of the roots and replanting, the prognosis is not at all favorable. When due to the last stages of pulpitis, misapplication of arsenic, the chances of recovery for the tooth are smaller.

Before noting the diseases of the maxillæ, it will be well to allude briefly to a disease of the alveoli, pyorrhea alveolaris or Riggs' disease. We are all very well aware of how little is known on this subject, and of the theories advanced. Why should we classify this as a special disease all by itself, instead of following a more logical course and regarding it as a pure periosteal one, for its pathology can be best understood if it is regarded as a form of osteo-periostitis, even though it is confined to the alveolus—

in fact a final stage of periostitis, as the effect upon the teeth is entirely secondary and consequent upon their separation from the soft parts. In its symptoms and characters, both macroscopically and microscopically, the course it follows and its amenability to various forms of treatment, it presents no special phenomena to distinguish it from simple caries of bone, save that the latter disease is almost always associated with an impaired condition of the general health. In this respect, and in the absence of fever, pyorrhea alveolaris more closely resembles Sir James Paget's *osteitis deformans*, concerning which I quote the following: "The surrounding gum becomes spongy, deep red, and sometimes tender; it separates from the neck of the tooth, while at the same time the periosteum suppurates and discharges pus, which is continually oozing out around the necks of the teeth and can be generally pressed out in great quantities. It is extremely chronic, begins generally in early middle age, and may continue for an indefinite time without influencing the general health. The early stages of the disease are sometimes attended with pain varying widely in severity. The breath is usually foul, the roots covered with irregular granular masses of greenish or blackish tartar, the discharge offensive and the whole mouth tender." The causes are not easy to find in all cases, but they are similar to those which cause periostitis. Injury of any sort to the periosteum, as cold, a blow, or excessive bite; a strumous or syphilitic diathesis seems often to co-exist.

The treatment should be based on surgical and medical principles, allowing for location, exactly like caries of bone, where the use of steel instruments is usually contra-indicated on account of the further injury which is likely to ensue. Unfortunately, our knowledge has been handicapped thus far by regarding this disease as a purely dental one, confined to dental tissues, instead of a surgical bone disease. The more the disease was regarded as dental, the more did we forget that the alveolus was only ordinary bone, subject to the same changes as any other bone, and having just as unsatisfactory treatment as caries has wherever it occurs.

### NECROSIS CONTRASTED WITH CARIES.

| NECROSIS. | CARIES. |
| --- | --- |
| 1. *Parts affected.*—In compact tissue. Blood vessels are better supported in compact bone and so less liable to passive congestion, but from the narrowness of the canals they are quickly strangulated by the pressure of the exudation, and so the bone is rapidly and completely deprived of vitality. | 1. Most common in cancellous tissue. Here is room for dilatation and exudation without their causing a sudden stasis in the vessels. |
| 2. *Result of Probing.*—A probe is suddenly arrested by striking against hard bone, without giving rise to pain. | 2. It is felt to pass through soft inflamed bone and this is quite sensitive. |
| 3. *Nature of the Discharge.*— Is mostly purulent. | 3. More watery or serous and has a greater amount of lactic acid. |
| 4. *Granulations along the sinus and at its orifice.*—Are comparatively healthy, often fungous and florid. | 4. Small; or large, pale and edematous. |
| 5. *Cause.*—The more acute, local injury or constitutional, as acute specific fever, the more likely is inflammation to end in necrosis. | 5. In scrofula, caries is most common. |
| 6. *In syphilis.*—Both are frequent. | |

Under the head of hyperostosis we will group cases in which general enlargement of the maxillary bones occurs, without any tumor which could be properly placed among the osteomata. Enlargements of the angles of the inferior maxilla, quite apart from the development of the teeth and giving a peculiarly broad appearance to the face, occur in otherwise healthy subjects of about twenty, and they appear to be stationary. In true hyperostosis, however, there are always large nodules of bone, often symmetrical, thrown out by the bones of the face and cranium, which slowly but steadily increase in size, producing hideous deformity and finally causing death. Heath has operated on some cases where the disease was unilateral, with good success.

Cysts occur in both jaws, either single or multiple. Their origin is probably in the cancelli of the bone, and is in many cases due to irritation caused by neighboring teeth; a cancellous being filled with fluids expands, and produces a gradual absorption and obliteration of neighboring cancelli until a cyst of considerable size is formed.

The multilocular cysts of the inferior maxilla appear to be more closely connected with the teeth than the single cysts, since in many cases the extraction of teeth or stumps gives exit to a quantity of glairy discharge. Distension and absorption of the alveoli go on as the cysts increase in size, so that the walls at length become membranous and the macerated bone shows great gaps in outline. A remarkable point is the length of time over which they may extend without injuring the patient except by inconvenience.

[*To be Continued* ]

